# Quantitative evaluation of a single-distance phase-retrieval method applied on in-line phase-contrast images of a mouse lung

**DOI:** 10.1107/S1600577514009333

**Published:** 2014-05-16

**Authors:** Sara Mohammadi, Emanuel Larsson, Frauke Alves, Simeone Dal Monego, Stefania Biffi, Chiara Garrovo, Andrea Lorenzon, Giuliana Tromba, Christian Dullin

**Affiliations:** a The Abdus Salam International Centre for Theoretical Physics, Trieste, Italy; b Synchrotron Light Source ‘Elettra’ Trieste, Strada Statale 14, km 163.5 in AREA Science Park, Basovizza 34149, Italy; c Department of Physics, Chemistry and Biology, Linköping University, SE-581 83, Sweden; d Department of Architecture and Engineering, University of Trieste, Trieste, Italy; e Department of Hematology/Oncology, University Hospital Goettingen, Robert Koch Strasse 40, Goettingen, Lower Saxony 37075, Germany; f Cluster in Biomedicine s.c.r.l., AREA Science Park, Strada Statale 14, km 163.5, Basovizza, 34149 Trieste, Italy; g Institute for Maternal and Child Health, IRCCS Burlo Garofolo, via dell’Istria 65/1, 34137 Trieste, Italy; h Department of Diagnostic and Interventional Radiology, University Hospital Goettingen, Robert Koch Strasse 40, Goettingen, Lower Saxony 37075, Germany

**Keywords:** computed tomography, phase-contrast imaging, phase retrieval, lung imaging

## Abstract

Propagation-based X-ray phase-contrast computed tomography (PBI) has already proven its potential in a great variety of soft-tissue-related applications including lung imaging. However, the strong edge enhancement, caused by the phase effects, often hampers image segmentation and therefore the quantitative analysis of data sets. Here, the benefits of applying single-distance phase retrieval prior to the three-dimensional reconstruction (PhR) are discussed and quantified compared with three-dimensional reconstructions of conventional PBI data sets in terms of contrast-to-noise ratio (CNR) and preservation of image features. The PhR data sets show more than a tenfold higher CNR and only minor blurring of the edges when compared with PBI in a predominately absorption-based set-up. Accordingly, phase retrieval increases the sensitivity and provides more functionality in computed tomography imaging.

## Abstract 2

Quantitative analysis concerning the application of a single-distance phase-retrieval algorithm on in-line phase-contrast images of a mouse lung at different sample-to-detector distances is presented.

## 1. Introduction   

Within the aim of unravelling the patho-mechanism of lung disease and the testing of novel treatments, there is still a strong need for improvement of lung imaging techniques and their application in preclinical disease models. Owing to the very nature of the lung with its air–tissue interfaces, lung imaging remains challenging for most imaging modalities (Kauczor & Kreitner, 1999 ▶). Propagation-based phase-contrast computed tomography (PBI) has already been proven valuable in applications focusing on low-absorbing tissue (‘soft tissue’) (Kitchen *et al.*, 2005 ▶; Sera *et al.*, 2005 ▶). The obtained edge effects facilitate the delineation of the airways, but on the other hand hamper or prohibit further quantitative analysis relying on threshold-based segmentation of the data sets. To circumvent this problem, edge-suppression techniques or low-pass filters can be used to remove these effects. However, this also diminishes the quality of the image features, especially for edges. Here, we propose and validate the application of a single-distance phase-retrieval method (Paganin *et al.*, 2002 ▶) for in-line phase-contrast computed tomography (CT) imaging of a mouse lung *in situ* filled with air at a physiological pressure. Several other phase-retrieval techniques utilizing multiple sample-to-detector distances (Mayo *et al.*, 2012 ▶; Kostenko *et al.*, 2013 ▶; Cloetens *et al.*, 1999 ▶) have been utilized before but are not practical for several reasons: *e.g.* they need an advanced imaging set-up and are very sensitive to variations in the incident beam, an aspect which needs to be considered at synchrotron light sources. Additionally, the movement and differences in the total amount of optical energy between projections acquired at different distances causes slight shifts and results in further alterations and artefacts. More importantly, multiple measurements increase the exposure time and dose delivered to the biological sample. Therefore, a single-distance phase-retrieval algorithm based on the transfer of intensity equation (Paganin *et al.*, 2002 ▶; Gureyev *et al.*, 2009 ▶; Teague, 1983 ▶), which only requires one CT data set obtained at a single sample-to-detector distance, has many advantages. This algorithm reconstructs the three-dimensional distribution of the complex refractive index, 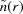 = 1 − 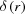 + 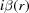, inside the sample by using X-ray projection images acquired at different view angles (rotational position of the sample). This class of algorithms can strictly speaking only be applied for ‘homogeneous’ objects, which are either pure absorption, pure phase objects or objects characterized by a constant ratio of the real to the imaginary parts of the refractive index, *i.e.* 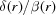 = 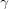 = a constant (Paganin *et al.*, 2002 ▶). Although biological samples do not satisfy this assumption, it has been demonstrated by Wu *et al.* (2005 ▶) that these kinds of algorithms can still be applied for biological samples which are predominately composed of materials with a low atomic number (*Z* < 10), referred to as ‘soft tissue’ within this article. Here we use an *in situ* mouse lung sample and show that, even in the presence of material with *Z* > 10, such as bone, the image quality can be dramatically increased by single-distance phase retrieval and exceeds that of PBI.

## 2. Methods   

### 2.1. Sample preparation   

The chest imaged in this study was taken from a mouse sacrificed using a xylazine–tiletamine–zolazepam overdose. In order to reproduce conditions which resemble the *in vivo* situation, the lung was inflated *in situ* with air, under a constant pressure of 30 cm water column (2.94 kPa), through a series of smaller tubings, down to a polyethylene cannula (PE50) fixed inside the trachea with a cotton wire. To block the air inside the lung the trachea was tied up. Following this procedure the sample was kept at room temperature for 2 h, in order to avoid any ‘rigor mortis’-based alterations. In the final step the sample was embedded in 1% agarose gel, inside a 30 ml falcon tube (Fisher Scientific, USA) serving as a sample holder, thus avoiding air leakage, alterations and movement during the time course of the X-ray examination. The agarose gel was left to set for another 30 min at 277 K, which allowed for a complete gelatinization of the gel. Following this procedure the sample was placed inside the SYRMEP beamline experimental hutch 30 min before imaging, thus allowing for temperature adaptation in order to suppress any alterations of the lung during the scanning process.

### 2.2. Data acquisition   

The chest area of the sample was scanned at the SYRMEP beamline of the Elettra synchrotron light source (Trieste, Italy). The sample was scanned at three sample-to-detector distances of 7 cm, 30 cm and 100 cm, with the following parameters: X-ray energy = 22 keV, with a spatial resolution of 9 µm; field of view of 18 mm × 12 mm; 1800 projections over a full rotation of 360°.

### 2.3. Phase retrieval   

Owing to phase contrast, the obtained projection images display a mix of absorption-based contrast and edge effects whose magnitudes depend on the sample-to-detector distance. In order to enable threshold-based segmentation and to fully exploit the potential of phase-contrast CT, it is necessary to calculate an image which is predominated by the real part of the complex refractive index and without the edge effects. Therefore, to reconstruct the complex refractive index 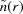 in the sample, a single-distance phase-retrieval algorithm, based on the transfer of intensity equation (TIE), is applied to the acquired data sets (Paganin *et al.*, 2002 ▶). Furthermore, only one scan per sample is needed, thus enabling an overall scanning time of about 1.5 h in a 360° mode and therefore reduces artefacts based on alterations of biological samples over time. We used a TIE phase-retrieval algorithm implemented in the *X-Tract* software package developed at CSIRO (Paganin *et al.*, 2002 ▶).

For this algorithm *a priori* knowledge of the ratio (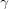) between 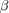 and 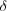 of the refractive index is needed. Here we used 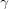 = 1950 for lung tissue. This value is based on the standardization of lung tissue by the International Commission on Radiological Protection (ICRP) which is described by hydrogen, carbon and oxygen in the following ratios: H 10, C 0.83, O 5 (Berger, 1992 ▶). This soft-tissue equivalent was used in the online calculator for the refractive index (Center of X-ray Optics, Lawrence Berkeley National Laboratory, http://henke.lbl.gov/optical_constants/getdb2.html) to obtain γ. 

To evaluate the benefit of single-distance phase retrieval over conventional PBI, slices were also reconstructed without prior application of a phase-retrieval algorithm.

### 2.4. Post-processing and quantification   

All scans were reconstructed after application of the TIE phase-retrieval algorithm (PhR) and without phase retrieval (PBI). For quantitative analysis the PBI and PhR data sets were registered to the PBI 7 cm data set using a two-dimensional cross-correlation evaluation in order to identify the corresponding slice and a Fourier–Mellin algorithm to detect in-plane scaling, rotation and translation (Zitová & Flusser, 2003 ▶). The two-dimensional cross correlation used as a measure for similarity between two images was strongly influenced by the edge effects in the PBI data sets and prevented the Fourier–Mellin algorithm from converging. Therefore, the PBI data sets were filtered only for the registration process by using a normal mean filter (size 3 × 3 × 3 voxels) to suppress the edge effects. Profiles at air–tissue interfaces were calculated to assess the edge quality in the images. Standard deviations and mean values were measured in different volumes of interest (0.4 mm^2^) for fat, air, soft tissue and bone in all data sets. The contrast-to-noise ratio (CNR) between two adjoined tissues was calculated based on equation (1) (Muhogora *et al.*, 2008 ▶).

In order to assess the quality of the edges in the images, the edge-enhancement index [EEI; equation (2)] (Donnelly *et al.*, 2006 ▶) was calculated. However, the highest and lowest value (*P* and *L*) on a profile plot used in the proposed equation (2) by Donnelly *et al.* is difficult to define within the sometimes sparsely sampled line profile, especially in the presence of the edge effects of the PBI images. Thus, we introduced a measure based on a non-linear fit of a sigmoid function [equation (3)] on these profiles. In order to avoid the registration process from influencing the noise level determination, the noise level was measured in the original untransformed data.
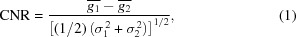
where 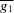 and 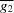 are the mean intensity values of a given homogenous area (size 0.4 mm^2^) in tissue and in air, respectively, and 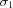 and 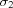 are the corresponding standard deviations;
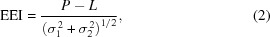
where *P* and *L* are the highest and lowest values on a profile plot of an air–tissue interface (length 0.1 mm), and 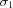 and 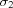 are the standard deviations of the profile regions depicting pure air and pure tissue, respectively;
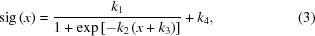
where the different constants *k*_*i*_ are used to adjust the sigmoid function to the present line profile. The steepness of the edge is depicted by the constant *k*_2_.

## 3. Results   

### 3.1. Overall performance of the used phase-retrieval algorithm   

**Figure 1:**
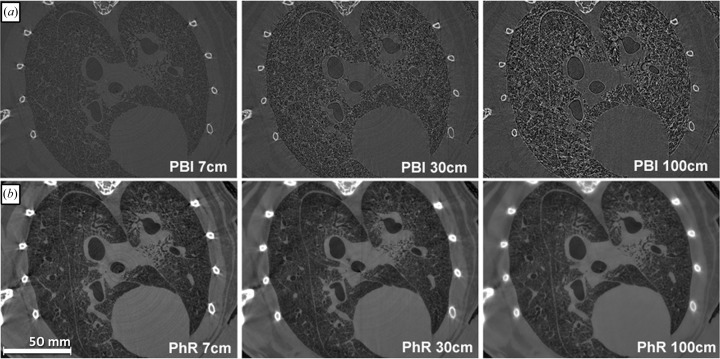
Comparison of the edge effects within the PBI data sets (*a*) at 7, 30 and 100 cm sample-to-detector distances. A clear increase of the edge effects can be seen with increasing distance. (*b*) The respective results from the TIE phase-retrieval algorithm (PhR) with a δ-to-β ratio optimized for lung soft tissue, therefore bone structures appear blurred. Besides a slight increased blurring at 100 cm the images look alike and present a higher contrast than the PBI data sets. Note that only the central part of the data is shown for convenience. The full reconstruction represents the entire cross section of the sample.

With increasing sample-to-detector distance the filtered back-projection (FBP) reconstruction of the PBI data sets reveals higher magnitudes of phase effects (Fig. 1*a* ▶). Phase retrieval is meant to calculate the δ-distribution (real part) of the complex refractive index within the sample and should therefore be independent of the sample-to-detector distance. The PhR results (Fig. 1*b* ▶) in general show the expected behaviour apart from a slight increased blurring, thus indicating the successful application of the used algorithm.

### 3.2. Quantitative comparison of the reconstructed phase-retrieved data sets with the PBI data sets   

In general, quantitative comparison is hampered by the fact that these two image types represent different features of the studied object: absorption plus edge enhancement in the PBI data sets, and phase-shift-dominated contrast without edge effects in the PhR data sets. Both can be advantageous in terms of the application of different image-processing protocols. Therefore, the following analysis is mainly meant to show the feasibility of using these data for threshold-based image segmentation or for visual inspection of the images. Thus, both contrast but also the quality of the edges of image features need to be addressed. The contrast is a measure of the effectiveness to discriminate between adjacent tissues and is positively dependent on the difference in the tissues’ respective grey values and negatively influenced by the noise level. In order to quantify the image contrast and account for the presence of noise, the contrast-to-noise ratio [equation (1)] was measured.

The same two-dimensional regions solely containing air or soft tissue (three different regions each, measured on six slices) with a size 0.4 mm^2^ were identified and their mean values and standard deviation were calculated in all data sets. These regions were selected away from tissue interfaces as to not be affected by the edges effects. The calculated CNR values show a high CNR between air and soft tissue within the lung of up to 29 for the phase-retrieved data set at 100 cm sample-to-detector distance (Table 1 ▶). Given the fact that the CNR increases with increasing sample-to-detector distance, this implies that imaging at higher distances may further enhance the results.

**Figure 2:**
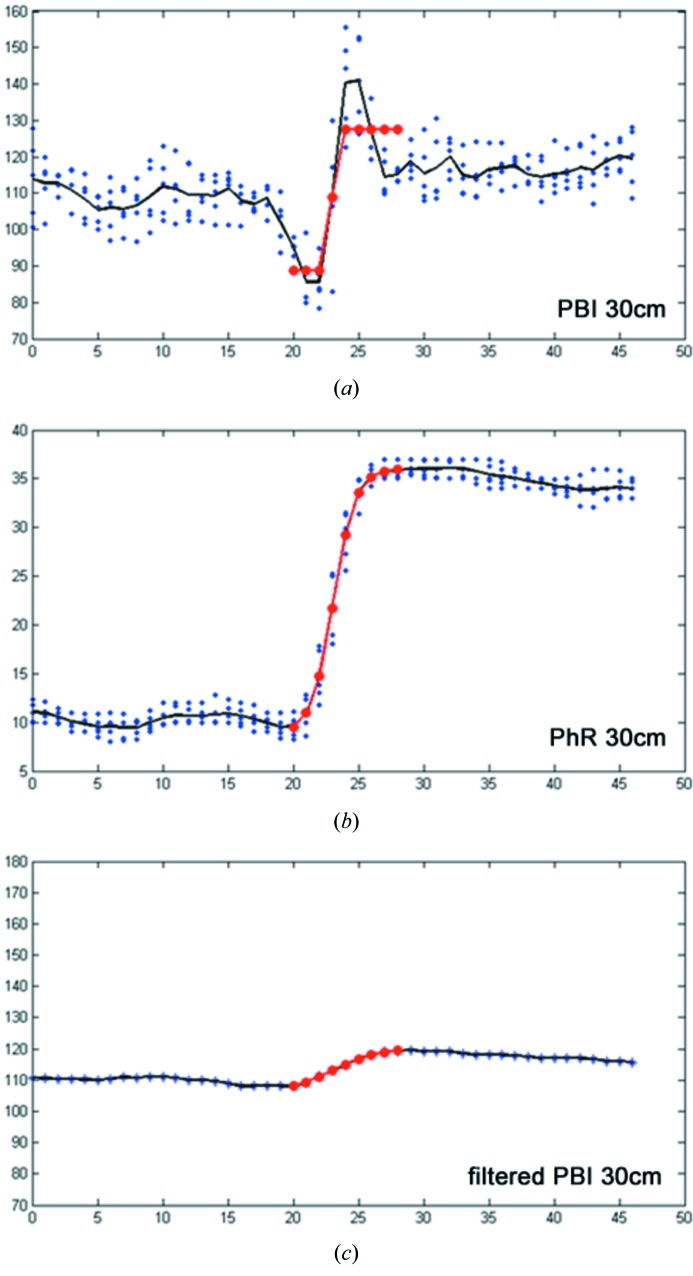
Comparison of the edge quality between PBI (*a*), PhR (*b*) and filtered PBI data sets (*c*). (*a*)–(*c*) show profile plots at the same location in PBI and PhR data sets obtained with a 30 cm sample-to-detector distance. In order to suppress the influence of noise, five individual profiles (blue dots) were measured and the average profile (black line) was used for evaluation. The red line resembles the fit of the sigmoid function [equation (3)]. A clearly steeper edge is apparent in (*a*) due to the strong edge effects; (*b*) shows a smoother edge but in combination with reduced noise; (*c*) presents the profile of the PBI data set after iterative use of a low-pass filter to reach the same edge steepness as in (*b*).

Additionally, five line profiles (0.2 mm length) at an air–soft-tissue interface were measured and the average of these line profiles was used to analyse the edge quality using equation (2) (EEI). As shown in Table 1 ▶, the EEI values for the PhR data sets are higher due to the strongly reduced noise of the profile. Therefore, EEI cannot reflect the true situation displayed in Fig. 2(*a*) ▶ compared with Fig. 2(*b*) ▶ which shows a much steeper and higher edge due to the edge effects in the PBI rather than in the PhR data sets.

Therefore, in order to quantify and compare the steepness of the edges and the influence of the edge effects we used a non-linear fitting regime for the measured profiles utilizing a sigmoid function [equation (3)] (Fig. 2 ▶, Table 1 ▶). Based on this equation the parameter 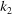 reflects the steepness of the edge. In order to provide more intuitive values, the highest 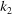 value (PBI 30 cm) was set to 100% and all the other values were expressed as a ratio of this reference value (steepness-of-fit). In contrast to EEI, the steepness-of-fit parameter reflects the observed increase in blurring in the phase-retrieved data sets and shows a slight decrease from 8% for 7 cm to 5% for 100 cm. This behaviour will hamper the use of very large sample-to-detector distances at least if a high spatial resolution in the range of the pixel size of the detection system is needed. Owing to the strong edge effects in the PBI data sets the edges appear steeper compared with the PhR data sets, ranging from 57% for 7 cm to 100% at 30 cm. The breakdown in the edge steepness at 100 cm in the PBI data is caused by phase effects produced by the tissue texture, which carries more weight at greater sample-to-detector distances, and by the appearance of higher-order interference fringes, which cannot be properly sampled with the limited detector pixel size of 9 µm (binning 2 × 2 used in this study). These effects create massive distortion of the measured edge profile which prevents the applicability of a fitting approach with a sigmoid function [equation (3)] and therefore diminishes the measured edge-steepness.

### 3.3. Does phase retrieval do more than a low-pass filter applied to the raw data sets?   

As shown in Fig. 2(*a*) ▶, PBI at 30 cm is characterized by strong edge effects and therefore presents very steep edges compared with the phase-retrieval data set PhR of the same sample-to-detector distance (Fig. 2*b* ▶). In order to prove that phase retrieval cannot be substituted by a simple low-pass filter to remove the edge effects, the PBI data set was gradually filtered using a Gaussian filter (kernel with 3 pixels) until the profile presented the same steepness as the PhR data set. Even in this ideal situation where the CNR is increased due to the suppressed noise, it only reaches about 5 as against 17.5 obtained with the PhR data set. This indicates that phase retrieval cannot be substituted by low-pass filtering.

### 3.4. Single-distance phase-retrieval applied to in-line phase-contrast synchrotron-radiation-based CT data sets of an *in situ* mouse lung opens up for structural analysis of lung tissue   

**Figure 3:**
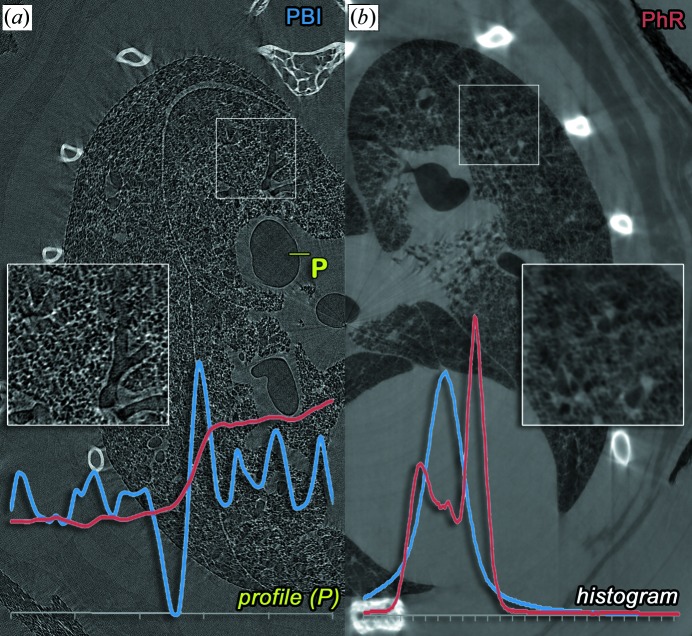
A direct comparison of the image features for PBI (*a*) and the TIE phase-retrieved data set (*b*) is shown, both obtained for the same sample at an object-to-detector distance of 30 cm (this figure depicts only the central part of the reconstruction for convenience). As indicated in the detailed views, PBI presents strong edge effects when compared with PhR. The overall grey value histograms (lower right corner) for PBI (blue) and PhR (red) show that, due to the phase effects overlaying the measured absorption, only one Gaussian-shaped peak can be seen. Therefore, no intensity-based segmentation between air and tissue can be performed. In contrast, after phase retrieval the histogram is clearly composed of two density intervals. The line profile at the position P in PBI (blue) and PhR (red) in the lower left-hand corner shows large variations and a strong edge effect for PBI, whereas in PhR the profile resembles the expected jump-function for a simple air–tissue interface. These drastic edge effects also cause negative values lower than the value for air, within the bronchi (dark contours). Therefore, the airways may appear filled, which is not the case. Note that the δ-to-β ratio for PhR was optimized for soft tissue and did not match the ratio of bone, which is why ribs and spine appear more blurred than in PBI.

Utilizing the TIE phase-retrieval algorithm we have reached a more than ten times higher CNR value in the images of an *in situ* mouse lung. Fig. 3 ▶ exemplifies the difference in the appearance of PBI and PhR data sets by showing the same slice at 30 cm cut in the middle. In Fig. 3(*a*) ▶, PBI depicts the clear delineation of the air to soft-tissue interface and the presence of strong edge effects. The blue line in the profile plot at position P shows the large variation of the grey values and the strong edge effect at the interface. In addition, the overall histogram of this slice allows no contrast-based separation of tissues, presenting only one Gaussian-shaped distribution [Fig. 3(*b*), blue histogram]. In contrast, the PhR data set in Fig. 3(*b*) shows no signs of edge effects. The profile plot (red) depicts a common stair-shaped function with low variation within the air and the soft-tissue plateau phase. The histogram clearly shows at least two components for air and soft tissue which enables threshold-based segmentation and therefore quantitative image analysis.

## 4. Discussion   

Here we present the benefits of utilizing in-line phase-contrast CT for lung imaging in combination with single-distance phase retrieval as demonstrated on an *in situ* mouse lung sample. The application of in-line phase-contrast CT on lungs exploits the presence of the air–tissue interfaces and provides significantly better delineation of the airways than several other applications (Siu *et al.*, 2008 ▶; Sera *et al.*, 2005 ▶; Kitchen *et al.*, 2005 ▶). On the other hand, the strong edge effects in the data sets hamper the segmentation of different components (air, soft tissue) and therefore prevent a quantitative analysis.

By utilizing single-distance phase retrieval, as demonstrated in this study, data sets can be generated which predominately show the distribution of the real part of the complex refractive index within the samples and do not display any edge effects. We verified the reliability of this approach by analysing the same lung sample at different sample-to-detector distances and obtained the same results with every distance. Remarkably, the CNR of the generated data sets are more than ten times higher than with the classical absorption-based mode (short sample-to-detector distance). It has to be stated that the ten-fold gain in CNR is related to many different factors, including the overall set-up of the experiment, the characteristics of the chosen sample, the sample-to-detector distances, the resolution of the used detector system, characteristics of the incident X-ray beam, the used implementation of the reconstruction and the phase-retrieval software. Therefore, the calculated factor of ten does not represent a general rule when comparing phase-retrieved images with PBI images and may vary in other set-ups. Beltran *et al.* for instance reported a 9–200-fold increase in CNR (Beltran *et al.*, 2011 ▶).

In addition, CNR is an image parameter, which can be easily increased by de-noising. This usually suppresses high spatial frequencies and therefore diminishes the quality of edges. Therefore, it should not be used for quantification of image quality without a measure of the preservation of image sharpness. We repeatedly applied mean filtering on the PBI images to reach the same CNR as measured by PhR, but observed a dramatically lower sharpness of the edges than in PhR. This demonstrates that single-distance phase retrieval cannot be substituted by filtering of PBI images.

Interestingly, in the normal PBI data sets we also observed a decrease in CNR with increasing sample-to-detector distance. This is in contrast to our previous findings from the analysis of a phantom filled with different substances and imaged with two sample-to-detector distances (Gureyev *et al.*, 2013 ▶). We believe the loss in CNR is caused by the intrinsic small density variation within biological tissue, such as the lung. Even in areas solely composed of one tissue type, these variations cause additional phase effects which increase the image ‘noise’ and therefore diminish the CNR. This notion is supported by Donnelly *et al.* (2003 ▶), who quantitatively analysed the dependency of the observed phase effects of certain systemic parameters and found a strong impact of tissue texture and scattering on the detection of the phase effect fringes in biological samples. Our findings support these studies and underline the importance of evaluating novel imaging approaches in biological specimens.

Our data show that, even if biological samples do not fulfil the preconditions of a ‘homogeneous’ object (Gureyev *et al.*, 2009 ▶) for single-distance phase-retrieval algorithms and the generated data sets therefore predominately reflect only the real part of the complex refractive index, the achieved image quality outperforms that of absorption-based CT and PBI (phase-contrast CT without phase retrieval). In addition, the same short imaging time can be maintained with this single-distance phase-retrieval approach, something that would be impossible with other algorithms requiring multiple sample-to-detector distances. However, as previously reported (Beltran *et al.*, 2011 ▶), the application of this class of phase-retrieval algorithms requires *a priori* knowledge of the δ-to-β ratio of the refractive index of the analysed sample. In our study we accordingly chose the appropriate ratio for lung tissue (δ-to-β ratio = 1950). Therefore, the bone details, like the spine and rib cage, appear blurred in the reconstructions due to the fact that they are characterized by a δ-to-β ratio of about 250 (Center of X-ray Optics, Lawrence Berkeley National Laboratory, http://henke.lbl.gov/optical_constants/getdb2.html), based on the composition of bone of H 0.06, C 0.28, N 0.3, O 4.1, P 7, Ca 15 as found in the database of the National Institute of Standards and Technology (NIST). This underlines the fact that single-distance phase-retrieval algorithms cannot be used to calculate the δ-value distribution of the refractive index in samples with a strong variance of δ-to-β ratios.

Another interesting result is that the analysed CNR in the phase-retrieved data rises with increasing sample-to-detector distances. This suggests that setting up imaging beamlines with greater sample-to-detector distances may improve the quality of such a lung imaging approach even further. The measured gain in CNR directly translates into an increased sensitivity, which will allow for precise three-dimensional analysis of morphological alterations within, for instance, mouse lung disease models. We therefore believe that the method presented here can be beneficial in a wide variety of similar preclinical studies.

## Figures and Tables

**Table 1:** Quantitative results of the image comparison

	PBI			PhR		
	7 cm	30 cm	100 cm	7 cm	30 cm	100 cm
CNR air–soft-tissue	1.55 ± 0.23	0.92 ± 0.25	0.73 ± 0.25	9.33 ± 0.92	17.54 ± 1.77	29.29 ± 10.55
EEI	8.80 ± 1.39	12.45 ± 0.80	11.24 ± 2.07	25.41 ± 2.29	51.31 ± 5.01	63.03 ± 2.02
Steepness of fit	57%	100%	15%	8%	7%	5%
